# Heat Shock Protein 70 (Hsp70) Peptide Activated Natural Killer (NK) Cells for the Treatment of Patients with Non-Small Cell Lung Cancer (NSCLC) after Radiochemotherapy (RCTx) – From Preclinical Studies to a Clinical Phase II Trial

**DOI:** 10.3389/fimmu.2015.00162

**Published:** 2015-04-15

**Authors:** Hanno M. Specht, Norbert Ahrens, Christiane Blankenstein, Thomas Duell, Rainer Fietkau, Udo S. Gaipl, Christine Günther, Sophie Gunther, Gregor Habl, Hubert Hautmann, Matthias Hautmann, Rudolf Maria Huber, Michael Molls, Robert Offner, Claus Rödel, Franz Rödel, Martin Schütz, Stephanie E. Combs, Gabriele Multhoff

**Affiliations:** ^1^Radiation Oncology, Klinikum rechts der Isar, Technische Universität München, Munich, Germany; ^2^Transfusion Medicine, Institute for Clinical Chemistry and Laboratory Medicine, University Hospital Regensburg, Regensburg, Germany; ^3^Münchner Studienzentrum (MSZ), Klinikum rechts der Isar, Technische Universität München, Munich, Germany; ^4^Thoracic Oncology, Asklepios Lungenfachkliniken, Munich, Germany; ^5^Radiation Oncology, University Hospital Erlangen, Erlangen, Germany; ^6^GMP Laboratory, apceth GmbH & Co. KG, Munich, Germany; ^7^Thoracic Oncology, Klinikum rechts der Isar, Technische Universität München, Munich, Germany; ^8^Radiation Oncology, University Hospital Regensburg, Regensburg, Germany; ^9^Thoracic Oncology, Department of Medicine, University of Munich, Munich, Germany; ^10^Radiation Oncology, University Hospital Frankfurt, Frankfurt, Germany; ^11^Thoracic Oncology, Klinikum Bogenhausen, Munich, Germany; ^12^Institute of Biological Molecular Imaging, Helmholtz Zentrum München, Munich, Germany

**Keywords:** Hsp70-based immunotherapy, NSCLC patients, radiochemotherapy clinical trial, clinical phase II, NK cells

## Abstract

Heat shock protein 70 (Hsp70) is frequently overexpressed in tumor cells. An unusual cell surface localization could be demonstrated on a large variety of solid tumors including lung, colorectal, breast, squamous cell carcinomas of the head and neck, prostate and pancreatic carcinomas, glioblastomas, sarcomas and hematological malignancies, but not on corresponding normal tissues. A membrane (m)Hsp70-positive phenotype can be determined either directly on single cell suspensions of tumor biopsies by flow cytometry using cmHsp70.1 monoclonal antibody or indirectly in the serum of patients using a novel lipHsp70 ELISA. A mHsp70-positive tumor phenotype has been associated with highly aggressive tumors, causing invasion and metastases and resistance to cell death. However, natural killer (NK), but not T cells were found to kill mHsp70-positive tumor cells after activation with a naturally occurring Hsp70 peptide (TKD) plus low dose IL-2 (TKD/IL-2). Safety and tolerability of *ex vivo* TKD/IL-2 stimulated, autologous NK cells has been demonstrated in patients with metastasized colorectal and non-small cell lung cancer (NSCLC) in a phase I clinical trial. Based on promising clinical results of the previous study, a phase II randomized clinical study was initiated in 2014. The primary objective of this multicenter proof-of-concept trial is to examine whether an adjuvant treatment of NSCLC patients after platinum-based radiochemotherapy (RCTx) with TKD/IL-2 activated, autologous NK cells is clinically effective. As a mHsp70-positive tumor phenotype is associated with poor clinical outcome only mHsp70-positive tumor patients will be recruited into the trial. The primary endpoint of this study will be the comparison of the progression-free survival of patients treated with *ex vivo* activated NK cells compared to patients who were treated with RCTx alone. As secondary endpoints overall survival, toxicity, quality-of-life, and biological responses will be determined in both study groups.

## Introduction

The major stress-inducible heat shock protein 70 (Hsp70) is known as a cytoprotective molecular chaperone, which is frequently overexpressed in a large variety of tumor cells. As a molecular chaperone Hsp70 supports the correct folding of nascent and misfolded proteins, prevents protein aggregation following stress and assists protein transport across membranes (1). High cytosolic Hsp70 levels in tumor cells are associated with poor prognosis, metastatic spread (2) and resistance to standard therapies, such as radiochemotherapy (RCTx) (3–7). Inside tumor cells Hsp70 contributes to tumor cell survival by interfering with apoptosis pathways (8, 9).

Apart from these intracellular chaperoning functions Hsp70 has been found to be expressed on the cell surface of highly aggressive primary and metastatic tumor cells. This finding was not expected since Hsp70 lacks a transmembrane domain. Initially, the membrane expression of Hsp70 was proven by selective cell surface iodination (10) and by biotinylation followed by proteomic profiling of cell surface bound proteins (11). Later a mouse monoclonal antibody was established [cmHsp70.1 mAb; (12)], which is able to detect mHsp70 highly selectively on the plasma membrane of viable tumor cells by flow cytometry. Screening of viable single cell suspensions of more than 1,000 freshly isolated tumor biopsies and their corresponding normal tissues revealed that more than 50% of all tumors, but none of the healthy normal tissues exhibited a mHsp70-positive phenotype (13, 14). A prognostic value of mHsp70 on tumor cells has been demonstrated in xenograft tumor mouse models (13), since metastases of orthotopically implanted primary tumor cells showed a significantly higher surface Hsp70 density than the primary tumor cells (15). Moreover, survival of patients with mHsp70-positive squamous cell carcinoma of the lung and lower rectal carcinomas revealed a significantly decreased overall survival (OS) (16) compared to their mHsp70-negative counterparts.

Evidence is accumulating that cell surface translocation of Hsp70 in tumor cells is mediated via non-classical vesicular pathways (17) since inhibitors of the classical ER-Golgi transport route (Brefeldin A, Monensin) did not affect the cell surface expression of Hsp70. Anchorage of Hsp70 in the plasma membrane of tumor cells is most likely enabled by tumor-specific lipid components, such as globotriaosylceramide Gb3 (18), which directly interact with Hsp70 and are compounds of lipid rafts. Whether mHsp70 in lipid rafts mediates chaperone activity for cell surface signaling receptors is still a matter of debate. Following stress, such as a non-lethal radio- or chemotherapy not only the intracellular Hsp70 levels but also the membrane density of Hsp70 was found to be increased in tumor cells (19–21). Following irradiation Hsp70 is predominantly co-located with phosphatidylserine in the plasma membrane of tumor cells and thus has to leave the cholesterol-rich microdomain signaling platforms (22). Although elevated Hsp70 membrane and cytosolic levels confer resistance of tumor cells to standard therapies such as radio- and chemotherapy (2, 5, 6, 8), it also has been shown that mHsp70 serves as a target structure for activated natural killer (NK) cells. It appears that mHsp70 can fulfill dual functions: on the one hand, it can mediate protection against lethal damage, which is induced by radio- and chemotherapy; on the other hand, it might provide a target structure for the cytolytic attack by the innate immune system (13, 14, 16, 19).

## Preclinical Findings

### Activation of NK cells with Hsp70 peptide TKD plus IL-2 and identification of CD94 as a surrogate marker for cytolytic active NK cells

Previously, we demonstrated that incubation of NK, but not T cells, with peptide-free recombinant Hsp70 protein in combination with pro-inflammatory cytokines, such as IL-2 or Il-15 can stimulate the cytotoxic, proliferative, and migratory capacity of NK cells against highly aggressive, mHsp70-positive tumor cells, *in vitro* (14, 23). Similar to full-length Hsp70 protein, a 14-mer peptide (TKDNNLLGRFELSG, aa 450–463) also could activate the cytolytic and proliferative capacity of NK cells at equimolar concentrations (24). The stimulatory 14-mer peptide is an N-terminal extension of the 8-mer binding epitope of the antibody cmHsp70.1, which detects mHsp70 on the cell surface of tumor cells. Since the induction of the cytolytic activity of NK cells with the peptide is dose-dependent and saturable it is assumed that the interaction of NK cells with the peptide might be receptor-mediated. By antibody and protein/peptide blocking assays the C-type lectin receptor CD94 could be identified as a potential receptor, which mediates the interaction with the stimulatory Hsp70 peptide. CD94 forms a heterodimer either with the co-receptor NKG2A or NKG2C and thus acts as an inhibitory or activation receptor complex. Following incubation of NK cells with Hsp70 protein or Hsp70 peptide plus IL-2, the density of CD94 was found to be significantly up-regulated concomitant with an increased cytolytic activity against mHsp70-positive tumor cells (25, 26). Therefore, the density of CD94 on NK cells was considered as a surrogate marker for the cytolytic activity of NK cells against mHsp70-positive tumor cells.

### Mode of tumor cell killing of mHsp70-positive tumor cells by peptide plus IL-2 activated NK cells

It has been shown that cell membrane-bound Hsp70 renders tumor cells more susceptible to the lysis of NK cells that had been stimulated with Hsp70 protein/peptide plus low dose IL-2 (13, 14). In order to uncover the mechanism of lysis affinity chromatography, experiments were performed using lysates of activated NK cells on columns that were bound to either Hsp70 protein or Hsp70 peptide. Interestingly, the apoptosis-inducing serine protease granzyme B has been found to show an interaction with Hsp70 protein and peptide as determined by matrix-laser desorption ionization time of flight mass peptide finger printing (MALDI-TOF) (27). The interaction of granzyme B with Hsp70 was previously confirmed by Western blot and flow cytometry (27).

Natural killer cells that have been stimulated with Hsp70 plus IL-2 show a significantly up-regulated production of granzyme B in their intracellular vesicles. In contrast, the levels of perforin were found to be up-regulated only moderately (25, 26). Therefore, it is assumed that mHsp70-positive tumor cells are predominantly killed by granzyme B. Incubation of isogenic tumor cell systems that differ in their mHsp70 expression levels indicate that granzyme B in the absence of perforin effectively lysed mHsp70-positive tumor cells, but not their mHsp70-negative counterparts. Regarding these results, we concluded that Hsp70-positive tumor cells are killed by Hsp70 plus IL-2 activated, CD94-positive NK cells via granzyme B-mediated apoptosis (27).

### Preclinical models showing the efficacy of Hsp70 plus IL-2 activated NK cells

An incubation of purified human NK cells with Hsp70 peptide plus low dose IL-2 resulted in a specific tumor cell killing of mHsp70-positive, but not their mHsp70-negative counterparts, *in vitro*. Furthermore, activated NK cells compared to resting NK cells showed a significantly increased migratory capacity toward mHsp70-positive tumor cells as demonstrated in a transwell migration system. In order to proof the Hsp70-based anti-tumor activity of NK cells, *in vivo* immunodeficient SCID/beige mice bearing Hsp70-positive colon carcinoma cells were injected intravenous (i.v.) into the tail vein with either peripheral blood lymphocytes (PBLs), CD3-positively sorted T lymphocytes or CD3-negative CD94-positive NK cells that had been stimulated *ex vivo* with Hsp70 peptide plus low dose IL-2. A single injection of activated NK, but not of T cells, was found to result in a significant reduction in the tumor weight of the mice (28). After stimulation of PBL with Hsp70 peptide plus IL-2 activation markers such as CD25 or CD69 were found to be increased predominantly on the NK cell fraction. This result indicated that it is possible to selectively stimulate NK cells within unsorted PBL. Furthermore, the amount of tumor cell killing appeared to correlate with the actual number of activated NK cells that show a high expression density of CD94 (25, 26).

*Ex vivo* activated human NK cells could also control metastasized mHsp70-positive pancreatic carcinomas in immunodeficient mice, as shown previously (15). These data indicate that mHsp70 acts as a universal tumor-specific target structure, which is not restricted to only one specific tumor entity. In contrast, unstimulated NK cells did not induce tumor cell killing. Interestingly, NK cells that had been stimulated with IL-2 only were significantly less efficient in the control of the tumor growth in mice. A single injection of mice with Hsp70 peptide plus IL-2 activated NK cells, but not with identically stimulated T cells or IL-2 activated NK cells was also able to significantly enhance the OS of tumor-bearing mice (15). Following four repeated i.v. injections with Hsp70 peptide plus IL-2 pre-activated NK cells, the primary pancreatic tumor was found to be eliminated completely and hepatic metastases could be prevented (15).

## Clinical Results

### Summary of the findings of a phase I clinical trial using Hsp70 peptide plus IL-2 activated NK cells

**Figure 1:**
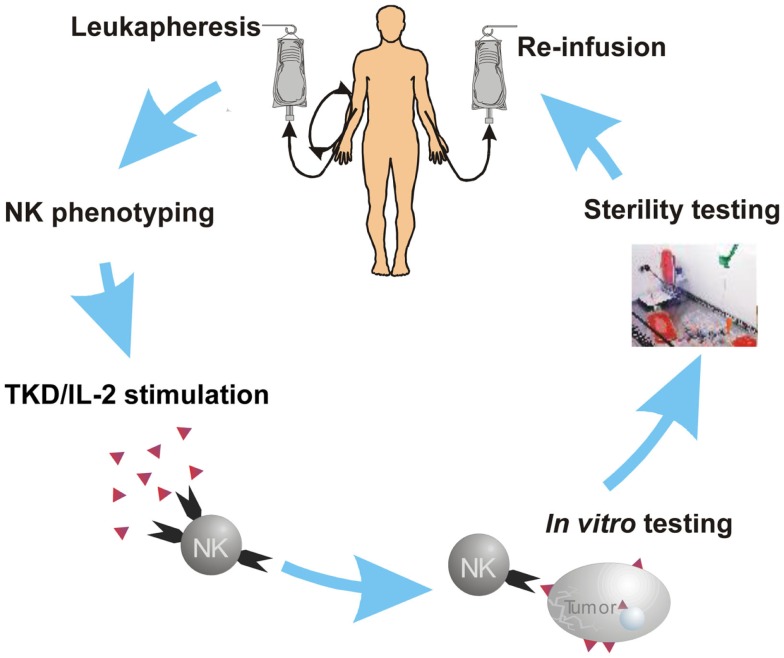
**Scheme of the NK cell activation in the clinical phase I trial**. NSCLC patients after radiochemotherapy undergo leukapheresis and the immune phenotype (NK cell and T cell markers) is assessed by flow cytometry. Following erythrocyte depletion on a SEPAX system peripheral blood lymphocytes (PBLs) are stimulated *ex vivo* in a GMP laboratory with TKD/IL-2 for 3–5 days. After measuring of NK activation markers and sterility testing, the activated cells are washed and re-infused (i.v.) in the patient.

Based on promising preclinical results using *ex vivo* activated NK cells in different tumor mouse models, a phase I clinical trial in tumor patients was performed in 2002 (29). Mouse models also have indicated that pre-activated NK cells are well tolerated even at high numbers (28). A major goal of the previously performed clinical phase I study (29) was to test whether a treatment of tumor patients with autologous, *ex vivo* Hsp70 peptide plus IL-2 activated NK cells is safe and well tolerated. After i.v. injection of escalating numbers of *ex vivo* pre-activated NK cells and escalating numbers of treatment cycles (up to six cycles) using complete leukapheresis products none of the patients showed any severe toxicities [no toxicities ≥grade 2 according to common toxicity criteria (CTC)]. Biological and clinical responses were evaluated in patients with confirmed metastatic colorectal cancer (*N* = 11) and non-small cell lung cancer (NSCLC) (*N* = 1) who failed clinical responses to standard therapies such as chemotherapy, radiotherapy, or laser-induced thermotherapy. At the beginning of the NK cell-based therapy, patients suffered from multiple pulmonary, hepatic, soft tissue and bone metastases, or local relapses. Because of the advanced tumor stages of the patients, it was ethically not accepted to obtain fresh biopsies to determine the Hsp70 status of the tumor during the study. Patients were enrolled in the study at least 4 weeks after the last standard therapy. Leukocyte concentrates were obtained from all patients by a 3–4 h leukapheresis at the Institute for Clinical Chemistry and Laboratory Medicine, University Hospital Regensburg. After sterile density gradient centrifugation lymphocytes were counted and re-suspended at cell densities of 5–10 × 10^6^/ml in fetal-calf serum (FCS)-free, GMP grade culture medium. For the dose escalation part, PBLs of the patients were frozen in aliquots. After simultaneous addition of Hsp70 peptide TKD (2 μg/ml, Bachem) and recombinant IL-2 (100 IU/ml Aldesleukine, Chiron) the cell suspensions were transferred into 250 ml Teflon culture bags (VueLife-118) and incubated in an incubator at 37°C for 3–5 days under gentle rotation. After washing in physiological saline (0.9% NaCl) and harvesting, the cells were suspended in physiological saline (500 ml) conditioned with 100 IU/ml recombinant IL-2. Sterility testing of the cell products was performed before, on day 3 and directly before re-infusion of the cells (Figure 1). Four of the 12 patients who were treated within an intra-individual and inter-individual dose escalation schedule showed no adverse effects. Therefore, from patient 4 onward all patients received the complete leukapheresis product containing between 0.7 × 10^9^ and 8.5 × 10^9^ PBLs in a single injection. The number of activated NK cells, which were re-infused ranged from 0.1 × 10^9^ to 1.5 × 10^9^ cells in all patients. These treatments were repeated up to six times in individual patients without observing any toxic side effects. The laboratory parameters, which were taken before and after each re-infusion cycle showed no NK cell-based treatment associated changes. A gradual deterioration of bilirubin, lactate dehydrogenase, and liver enzymes in some patients could be related to the disease progression. Irrespectively of the treatment cycle, the number of leukocytes, thrombocytes, and hemoglobin remained unaltered at each leukapheresis. After each re-infusion, vital parameters of all patients were monitored for at least 1 h. Only after the first re-infusion of the cells into the first patient, the patient was hospitalized overnight. A graphic overview of the NK cell activation process is shown in Figure 1. In step 1, patient received a leukapheresis, after gradient density centrifugation, patient-derived PBLs were tested for their activity by flow cytometry and functional assays. Then PBLs were incubated with TKD/IL-2 for 3–5 days in a GMP laboratory. After testing viability, sterility, and functional characteristics, cells were washed twice in 0.9% NaCl solution. Then activated cells were re-infused in 500 ml 0.9% NaCl conditioned with IL-2 (100 IU/ml) into the patient by i.v. injection within 30–60 min.

In addition to routine laboratory exams also specific laboratory parameters were determined from the blood product and from the patient before treatment, before leukapheresis, before cell re-infusion, and after cell re-infusion. The immune phenotype of the lymphocytes and the release of cytokines such as IFNγ, TNFα, and the apoptosis-inducing enzyme granzyme B were determined in the *ex vivo* cell culture before and after stimulation and in the blood of the patient before and after re-infusion of the activated cells. From day 1 to 4 of stimulation, the expression density of CD94 on activated NK cells continuously increased as it has been shown for healthy individuals previously. A comparison of the mean fluorescence intensity of the Hsp70 receptor CD94 after the first and the fourth treatment cycle also revealed a significant increase. These data indicate that similar to healthy controls the CD94 expression could also be increased on NK cells of tumor patients that had been treated with radio- and/or chemotherapy before. With respect to the cytolytic activity of *ex vivo* stimulated NK cells, 10 out of the 12 patients showed a significant increase after stimulation with Hsp70 peptide plus IL-2. In contrast, a stimulation of the patient’s cells with IL-2 alone showed no significant increase in the cytolytic activity. Studies using either Hsp70 specific or CD94 specific antibodies to block the target structure or the NK receptor demonstrated that Hsp70 is recognized on tumor cells by CD94-positive NK cells of the patients. Most interestingly, we could show that the cytolytic activity of *ex vivo* stimulated NK cells could be confirmed in the blood of the patients even 24 h after re-infusion of the cells. A comparison of the activity of NK cells in the blood before and after the fourth re-infusion cycle revealed significantly increased cytolytic responses of the blood lymphocytes in three out of five patients.

Clinical tumor responses (one stable disease, one mixed response) could be observed in one patient with colorectal and one patient with NSCLC who received at least four treatment cycles (29). Despite the low numbers, these findings were not expected due to the fact that all patients suffered from advanced tumor stages and had progressive disease during their last standard therapy.

In summary, the phase I clinical trial showed that re-infusion of Hsp70 peptide TKD plus IL-2 activated autologous NK cells is feasible, safe, and well tolerated. The immunological and clinical responses warrant additional studies in patients with a lower tumor burden and a confirmed mHsp70-positive tumor phenotype (29).

### Description of an ongoing proof-of-concept clinical phase II trial using Hsp70 peptide plus IL-2 activated NK cells in patients with NSCLC following radiochemotherapy

There is still a strong need to further improve the therapy of patients with non-resectable locally advanced NSCLC, since despite multimodal therapies the prognosis of those patients remains bad with a median OS of approximately 16 months. In this tumor stage, <20% of the patients survive more than 5 years (30). Several approaches to improve outcome have been evaluated, including systemic treatments or novel radiation techniques. The addition of chemotherapy did not enhance OS in these patients significantly (31). Although a distinct dose–response relationship is known for radiation therapy in lung cancer, escalated regimes have not improved outcome in NSCLC as shown in a prospective clinical trial (32). Immunotherapy seems to be a promising concept to improve the therapy of those patients. Since the membrane expression density of Hsp70 could be selectively enhanced on tumor cells following standard therapies such as ionizing irradiation and chemotherapy *in vitro*, we aimed to treat tumor patients with Hsp70 peptide TKD plus IL-2 activated (TKD/IL-2) autologous NK cells that had been treated with a RCTx. On the one hand, RCT should reduce the actual mass of viable tumor cells, and on the other hand, RCT should enhance the membrane density of Hsp70, which is recognized by pre-activated NK cells. Patients (*n* = 90) with NSCLC in non-metastasized but locally advanced stages IIIA and IIIB after RCTx (platinum based chemotherapy, 60–70 Gy) will be enrolled into the randomized multicenter clinical phase II trial (EUDRA-CT:2008-002130-30). Previous findings have indicated that a mHsp70-positive tumor phenotype is associated with a significantly decreased OS (16). Therefore, this interventional phase II trial incorporates a 1:1 randomized control group of patients that receive no adjuvant NK cell-based immunotherapy in addition to the current standard of treatment (simultaneous RCTx), and also exhibit a mHsp70-positive tumor phenotype.

**Figure 2:**
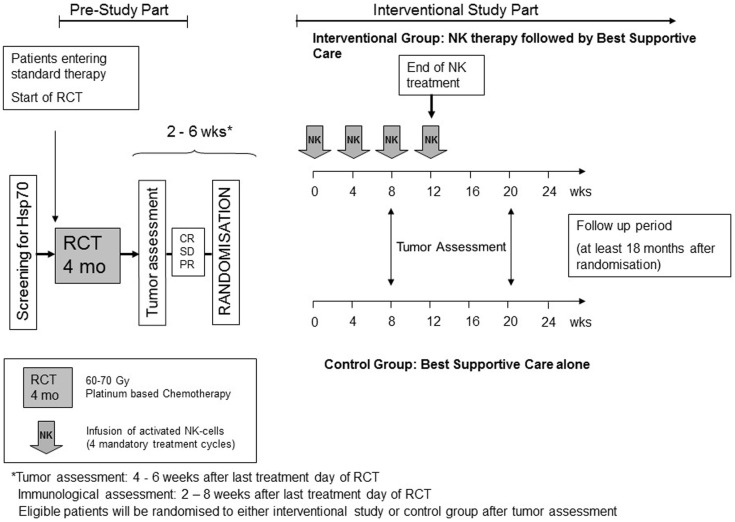
**Scheme of the phase II clinical trial**. In the pre-study part, the Hsp70 phenotype and the stage of the tumor disease is assessed. Only Hsp70-positive NSCLC patients in stage IIIA/B who received a radiochemotherapy (RCTx) and showed at least a stable disease are randomized into the trial. The interventional group receives four cycles of *ex vivo* TKD/IL-2 activated NK cells on a monthly basis; the control group gets best supportive care. Tumors will be assessed in the first year every 2–3 months by CT in both groups.

The major in- and exclusion criteria of the trial are summarized in Table 1. The scheme of the ongoing clinical phase II trial is shown in Figure 2. In a pre-study part, the Hsp70 phenotype of the tumor will be assessed in the blood of the patient by an Hsp70-specific ELISA and if available on tumor biopsies by flow cytometry using an Hsp70-specific mouse monoclonal antibody and the tumor stage will be determined. After successful RCTx (partial response, or at least stable disease) NSCLC patients in stage IIIA and IIIB will be randomized into the study. Patients in the interventional study arm receive four cycles of *ex vivo* TKD/IL-2 stimulated NK cells on a monthly schedule. Tumor assessment will be performed in both arms of the trial every 3 months for the first year and every 6-month thereafter until progression of disease.

**Table 1:** **In- and exclusion criteria of the ongoing phase II clinical trial: targeted NK cell-based adoptive immunotherapy for the treatment of patients with non-small cell lung cancer (NSCLC) after radiochemotherapy**.

Inclusion criteria	Exclusion criteria
First diagnose of histologically and/or cytologically proven and unresectable NSCLC with clinical stage III A and III B	Prior treatment with any other investigational drug within 4 weeks prior to first dose of study medication
Completion of radiochemotherapy no longer than 8 weeks ago	Any severe heart disease or any severe concomitant disease (ECOG status >2)
Progression free according to RECIST 1.1 criteria at the first assessment after completion of radiochemotherapy	NSCLC patients (stage IIIA/B) eligible for initial surgery with a confirmed consent of an interdisciplinary tumor board
Confirmed presence of Hsp70 on the patient’s tumor	Patients that show ALK positivity or an activating mutation of the EGFR-TK domain (assessment of ALK or EGFR status not mandatory)
Female or male, age 18–75 years	Patients with locally advanced or metastatic NSCLC other than predominantly squamous cell histology
ECOG status ≤2	Any disease (including psychotic disorders, drug abuse, active infection, uncontrolled hypertension, unstable angina, congestive heart failure, myocardial infarction within the previous year, serious cardiac arrhythmia requiring medication, hepatic, renal or metabolic disease), metabolic dysfunction, physical examination finding, or clinical laboratory finding like (in the investigator’s opinion) to affect the evaluation of the study or place the patient at risk whilst on treatment
Neutrophil count ≥1.5 × 10^9^/l after completion of radiochemotherapy	Any serious infection or sepsis
White blood cell (WBC) ≥2.5 × 10^9^/l after completion of radiochemotherapy	Any active autoimmune disease
Hemoglobin ≥8 g/l after completion of radiochemotherapy	Any immunodeficiency syndrome
Platelet count ≥100 × 10^9^/l after completion of radiochemotherapy	Surgery or immunotherapy within 4 weeks before study entry
Normal renal function (creatinine <150% ULN)	Patients with a known hypersensitivity to any of the administered substances should be excluded from the clinical trial
Normal liver function (bilirubin <150% ULN; G-GT, GPT and GOT <250% ULN)	Patients with a known positive HIV test should be excluded from the clinical trial as well as patients with positive hepatitis A, B, C tests (assessment not mandatory)
Normal blood coagulation (PTT 25–40 s)	Receipt of immunosuppressive drugs including systemic corticosteroids within 3 weeks before study entry. Low dose corticosteroids as they are a common treatment option for patients suffering from COPD are not an exclusion criteria
Measurable disease according to immune related RECIST criteria	Radio- cytostatic- and immunotherapy in parallel or within 2 weeks prior study start
Female patients of childbearing potential must have negative pregnancy test performed during screening period (≤14 days before initiation of study dosing)	Women who are pregnant or breast feeding
Postmenopausal women must be amenorrheal for at least 12 months to be considered of non-childbearing potential. Female patients of reproductive potential must agree to employ an effective method of birth control throughout the study and for 6 months following discontinuation of study drug	Female patients of reproductive potential unwilling to practice a highly effective method of birth control
Written (signed) informed consent document indicating that the patient of all pertinent aspects of the trial prior to enrollment and to participate in the study	History of non-compliance with medical regimes
Ability to comply with the study	Patients unwilling to or unable to comply with the protocol

The mHsp70 status on the tumor will be assessed in a pre-study screening part carried out in the Department of Experimental Radiation Oncology at the Klinikum rechts der Isar, Technische Universität München (TUM). Tumor biopsies obtained during the bronchoscopy at primary staging will be sent to the sponsor’s laboratory. Freshly isolated single cell suspensions of biopsies are used for analysis of the Hsp70 tumor phenotype. If bronchoscopy has been performed *alio loco* prior to patient admittance, the mHsp70 expression will be determined by quantifying the amount of exosomal Hsp70 in the blood of the patients. Previously, we have shown that mHsp70-positive tumor cells actively release Hsp70 in exosomes, which present Hsp70 on their surfaces. Since most commercial Hsp70 ELISAs are unable to detect lipid-bound, exosomal Hsp70 in the serum we established a novel lipHsp70 ELISA test. This ELISA detects and quantifies exosomal Hsp70 in the serum of patients with a high accuracy (patent filed). Using this Hsp70 ELISA, we could show that patients with tumors of different entities (including NSCLC patients) exhibit significantly higher Hsp70 serum levels than a group of age- and gender-matched healthy volunteers (33). Therefore, in the screening part of the study, Hsp70 serum levels will be measured in NSCLC patients before and after RCTx. In the therapeutic clinical phase II trial, Hsp70 serum levels will be assessed before and after NK cell-based immunotherapy for the treatment group and at identical fixed time points for the control group. The mHsp70 phenotype will be correlated with the Hsp70 serum levels and the tumor volume will be correlated with the Hsp70 serum level. Furthermore, the results of the Hsp70 serum values before and after therapy will help to elucidate, whether serum Hsp70 levels can improve the monitoring of the clinical outcome after therapeutic intervention. The results of the Hsp70 serum levels will also help to further validate the role of exosomal Hsp70 as a prognostic/diagnostic tumor biomarker, as it was suggested for head and neck, lung, colorectal, pancreatic, brain cancer, and leukemic patients before (33). Elevated levels of Hsp70 in the serum were also found in patients with squamous cell carcinoma of the head and neck (34) and glioblastomas (35).

Identical to the phase I clinical trial the leukapheresis products, which are used as source material for stimulated NK cells, are produced centralized at the Institute for Clinical Chemistry and Laboratory Medicine, Transfusion Medicine, University Hospital Regensburg, under GMP conditions in order to obtain comparable cell products. Afterwards cell processing is performed in a GMP cleanroom laboratory. For all manufacturing steps, the permission of the competent authorities was obtained. NSCLC patients in the treatment arm will be treated four times every 2–6 weeks with *ex vivo* TKD/IL-2 stimulated NK cells after RCTx. In case of significant toxicities, the treatment will be interrupted, the dose of re-infused cells will either be reduced or stopped. Patients in the control arm also have received standard RCTx prior to enrollment. Tumor assessment will be done for both study groups at enrollment, every 3 months during the first year and every 6-month thereafter until progression of disease. Response to treatment will be assessed centrally at TU München in order to prevent bias.

### Regulatory aspects and production of the investigational medicinal product of the phase II clinical trial (apceth GmbH und Co. KG, Munich)

The Investigational Medicinal Product (IMP) is classified as an advanced therapy medicinal product [ATMP, somatic cell product, Regulation (EC) No 1394/2007]. The process established in an academic institution for the phase I had to be transferred and adapted to the actual requirements for ATMP’s. The process required approximately 1.5 years and was completed with the manufacturer license granted to apceth by the local and national authority as a prerequisite for the clinical trial application by the sponsor. The manufacturing of TKD-activated autologous NK cells follows the principles of good-manufacturing practice (EU-GMP guidelines) in order to provide a robust and reproducible pharmaceutical product.

The implementation involved the development phase, the transfer to GMP, and the implementation of a GMP-compliant process. The development phase defined the process steps (closed systems wherever possible), the equipment and quality control (QC) methods, the definition of in-process controls, and the materials (should be available in adequate quality). The comparability to the process applied for phase I and the results obtained should be confirmed. The transfer phase involved a stepwise implementation to full GMP mainly relating to up-scaling from small-scale (microtiter-plates), medium-scale (bags with small volume of cell suspension), and large-scale in addition to the qualification of the materials and analytical methods. The GMP-process was established with the successful validation of the QC methods and the validation of the process by media-fills and qualification runs. The modifications to the phase I process relate to the exact specification of the starting material “apheresis product” including transport to the GMP-facility, the introduction of closed systems (density centrifugation of the starting material is now performed in a Sepax^®^ device and harvesting/washing procedures in closed systems; in a class B room), release criteria, and test methods. Special attention was paid to the reconstitution buffer [Ringer-lactate and 0.1% human serum albumin (HAS)] and the shelf-life (24 h) as the end product is delivered to several study centers. Most of the development work was attributed to the definition and validation of analytical QC methods (flow cytometry) and the qualification of ancillary and raw materials (for example, TKD and cytokines). Flow cytometry of the activated NK cell population was challenging and different approaches had to be evaluated resulting in a change of specification (CD94) and the introduction of a parallel culture for QC-testing to test for the mean fluorescence intensity. The test results according to the specifications for potency, purity, identity, and absence of microbial contamination/endotoxins/mycoplasma are the basis for the release of the final product for further application by the qualified person. This IMP is not cryo-preserved and has a limited shelf-life; at least the sterility results are not available at the time of application and requiring a well-defined aseptic manufacturing process.

The seven participating study sites have been initiated in 2014 and the study will last for approximately 2 years after the inclusion of the first patient (in 2015) into the interventional study part. Since the phase I trial (29) and pilot studies (36) have shown that four repeated infusion cycles of *ex vivo* stimulated, autologous leukapheresis products lead to elevated basal NK cell activity in the peripheral blood of the patients, four treatment cycles will also be administered in the clinical phase II trial. The NK cell activity in the peripheral blood of the patients will be determined prior to study entry and start of adjuvant immunotherapy, every 3 months after enrollment for the first year and every 6-month thereafter to determine the biological activity. Multiparameter flow cytometric analysis will be performed with the peripheral blood of the patients to determine the activation status of the NK and T cells using a pre-fixed panel of antibodies as indicated in Table 2. The cytotoxic response of patient derived NK cells before and after RCTx and after immunotherapy will be assessed by Europium cytotoxicity assays using K562 cells as a classical NK cell target and by measuring the density of NK/T cell activation markers such as CD94 and CD69 in the laboratory of Gabriele Multhoff at the TUM. Serum Hsp70 levels will be measured as mentioned above. In parallel to the blood analysis, tumor response assessment will be performed and centrally reviewed according to the immune related Response Criteria (irRC). Patients will be excluded when they show progressive disease according to irRC [increase of tumor burden more than 25% relative to nadir (minimum recorded tumor burden, confirmation by a repeat, consecutive assessment no <4 weeks from the date first documented)].

**Table 2:** **Panel of CE-certified, fluorescence-labeled antibodies (Beckman Coulter), which are used in the clinical trial**.

Fluorescence-conjugated antibodies	Article#	Specificity
IgG1-FITC/PE/PC7/APC	A07795/A07796	Control
	737662/IM2475	
IgG2a-PE/APC	A09142/A12693	Control
CD3-FITC^−^/CD94-APC^+^	A07746/B09980	NK cells
CD3-FITC^−^/CD56-PE^+^	A07746/A0788	NK cells
CD3-FITC^−^/CD16-PE^+^	A07746/A07766	NK cells
CD3-FITC^+^/CD56-PE^−^	A07746/A0788	NKT cells
CD3-PC7^+^	737657	T cells
CD3-PC7^+^/CD4-PE^+^	737657/A07751	T helper cells
CD3-PC7^+^/CD8-APC^+^	737657/IM2469	T cytotoxic cells
CD3-FITC^−^/CD19-PC7^+^	A07746/IM3628	B cells
CD3-FITC^−^/CD14-PE	A07746/A07764	Macrophages
CD45-APC^+^	A79392	Leukocytes
CD66b(CD67)-FITC^+^	IMO531-U	Neutrophils, eosinophils, granulocytes

*7-AAD (A07704) was used as a viability dye*.

## Concluding Remarks

This review aims to summarize results from bench to bedside experiments that resulted in the initiation of a phase II clinical trial using *ex vivo* activated NK cells in a targeted immunotherapy of NSCLC patients after RCTx bearing Hsp70 membrane-positive tumors. Hsp70 has been found to serve as a biomarker for highly aggressive tumors and metastases (5, 6, 19). Preclinical data have shown that stimulation of NK cells with Hsp70 peptide TKD plus IL-2 results in an increased migratory capacity of NK cells and an enhanced killing of Hsp70 membrane-positive tumor cells *in vitro* (24), and in relevant tumor mouse models (28). An increased expression density of the C-type lectin receptor CD94 has been identified as a useful surrogate for the cytolytic activity of NK cells (25, 26), and granzyme B-mediated apoptosis was found to be responsible for the killing of tumor cells presenting Hsp70 on their cell surface (27). A phase I clinical trial has shown that re-infusion of *ex vivo* TKD/IL-2 stimulated autologous NK cells in patients with late-stage colorectal cancers and NSCLC is feasible, safe, and well tolerated (29). As stress, such as RCTx has been found to increase the cell surface density of Hsp70 selectively on tumor cells (21), a proof-of-concept phase II clinical trial was initiated in NSCLC patients stage IIIA/B after RCTx.

## Conflict of Interest Statement

The authors declare that the research was conducted in the absence of any commercial or financial relationships other than indicated that could be considered as a potential conflict of interest.
